# Long-Term Outcome of Horses Undergoing Unilateral Mandibular Condylectomy and Meniscectomy for Temporomandibular Joint Disease

**DOI:** 10.3389/fvets.2022.898096

**Published:** 2022-05-02

**Authors:** Sarah A. White, Nathan C. Canada, James L. Carmalt, James Schumacher, Fernando N. Amitrano, Kyla Ortved, Travis J. Henry, Sabrina H. Brounts, Carolyn E. Arnold

**Affiliations:** ^1^Department of Veterinary Large Animal Clinical Sciences, Texas A&M University, College Station, TX, United States; ^2^Texas Equine Hospital, Bryan, TX, United States; ^3^Department of Large Animal Clinical Sciences, Western College of Veterinary Medicine, University of Saskatchewan, Saskatoon, SK, Canada; ^4^Department of Large Animal Clinical Sciences, University of Tennessee, Knoxville, Knoxville, TN, United States; ^5^School of Animal and Veterinary Sciences, Veterinary Clinical Centre, Charles Sturt University, Wagga Wagga, NSW, Australia; ^6^Department of Clinical Studies, New Bolton Center, University of Pennsylvania, Kennett Square, PA, United States; ^7^Department of Surgical Sciences, Large Animal Surgery, University of Wisconsin, Madison, WI, United States

**Keywords:** TMJ, TMD, osteoarthritis, septic arthritis, performance horse, athletic function

## Abstract

**Background:**

There are no reports describing the long-term outcome of sport horses undergoing unilateral mandibular condylectomy with meniscectomy (UMC) for treatment for severe temporomandibular joint (TMJ) disease (TMD). Whether horses undergoing UMC require a specialized diet, can return to riding with a bit, or return to intended function after surgery is unknown.

**Objective:**

To determine the long-term outcome of horses undergoing UMC for treatment of severe TMD.

**Study Design:**

A multi-institutional, retrospective study.

**Methods:**

Medical records obtained from seven equine referral hospitals of horses with severe TMD that underwent UMC were reviewed. Details regarding the presenting complaints, results of clinical examination, findings of diagnostic imaging, surgical technique, and outcome (including long-term follow-up with an owner questionnaire) were recorded.

**Results:**

Eleven horses fit the inclusion criteria. Three had severe idiopathic osteoarthritis, and eight had confirmed septic osteoarthritis of the TMJ. The most common post-operative complications were drainage and peri-incisional swelling (*n* = 5). One horse developed a hematoma at the surgical site because the facial artery was inadvertently transected during the approach, causing the condylectomy to be postponed. All horses were discharged alive from the hospital, and 10 returned to their previous or intended use. All had complete resolution of clinical signs of TMD. One mare was retired from athletic use due to her genetic value as a broodmare. One horse was euthanized 2 years after UMC due to progressively worsening of clinical signs of temporohyoid osteoarthropathy (THO), which were not present before surgery. When available, owner satisfaction of the results of the procedure was excellent.

**Main Limitations:**

Sample size; multiple institutions; owner bias.

**Conclusions:**

Unilateral mandibular condylectomy should not be considered a salvage procedure. Horses treated for severe TMD by UMC can return to their previous or intended level of athletic performance and do not require a specialized diet.

## Introduction

The temporomandibular joint (TMJ) of the horse is an incongruous joint formed ventrally by the mandibular condyle and dorsally by the zygomatic process of the temporal bone (1). The joint is divided into two compartments, which are separated by the fibrocartilagenous intra-articular disc and communicate only when one or both are diseased (2, 3). The discotemporal compartment is dorsal to the disc, and larger than the ventral discomandibular compartment. Disease of the TMJ can be determined by examining synovial fluid obtained by arthrocentesis, and by radiographic, ultrasonographic, computed tomographic (CT), and magnetic resonance imaging (MRI) (4).

Prospective clinical trials, systematic reviews, or multi-center reports of TMJ disease (TMD) in horses are lacking because of the rarity of the disease and subtlety or ambiguity of clinical signs associated with TMD (4–6). The belief that lameness, or specific ill-behaviors, such as head-tossing, bit refusal, lameness, or unwillingness to travel in a specific direction (5, 7, 8), are caused by pain in the TMJ has resulted in a variety of empirical or holistic treatments, such as massage therapy, acupuncture, chiropractic adjustments, or dietary supplementation (9). Reported clinical signs displayed by horses with TMD include difficulties with mastication (quidding or hyporexia); pain when opening the mouth; effusion or swelling of the TMJ; headshaking; head tilt; and refusal to accept a bit when being ridden (8, 10–14). Despite these reports, some studies have shown that horses with acute inflammation of the TMJ do not reliably quid, despite experiencing a change in the mechanics of mastication. This suggests that there may be gradations of inflammation, with clinical signs being exhibited when pain from TMD reaches an undetermined threshold (15).

Conditions affecting the TMJ of horses most commonly reported include septic arthritis (14, 16–18), osteoarthritis (7), fracture of the mandibular condyle (17), and luxation or subluxation of the joint (19). Treatment of horses for non-septic arthropathy of the TMJ usually includes systemic administration of a non-steroidal anti-inflammatory drug (20) or intra-articular injection of a corticosteroid, hyaluronic acid (21), or biological therapy (8, 22). The treatment for septic arthritis of the TMJ of the horse is the same for other joints in that it entails lavage of the joint, with or without arthroscopic debridement, and systemic and intra-articular antimicrobial therapy (11, 14). Unilateral mandibular condylectomy (UMC) with or without meniscectomy, has been reported as a treatment for horses suffering from trauma to the TMJ or osteoarthritis resulting from chronic infection (13, 16, 18, 23, 24). Follow-up of the horses of these reports was relatively short, ranging between 4 and 6 months, and therefore, the ability of horses that have undergone UMC to return to athletic performance is unknown. The objective of this study was to describe the clinical signs, results of diagnostic imaging, and long-term outcome of 11 horses with advanced TMD treated by UMC. The hypothesis was that UMC with meniscectomy would allow horses to be maintained on a normal diet and return to their intended function.

## Materials and Methods

The medical record database from seven equine referral centers were searched from January 2010 to December 2020 using the following keywords: temporomandibular joint, TMJ, mandible and condylectomy. Horses were included if they were diagnosed with TMD and were treated with UMC. The following data were obtained from the medical records: signalment, presenting complaint, duration of clinical signs, findings during physical examination, the TMJ affected (i.e., right or left), results of diagnostic imaging [radiography and computed tomography (CT)], results of bacterial culture and sensitivity testing, cytologic examination of synovial fluid, treatment prior to UMC, and details of the surgery. Additional data included complications experienced during or after surgery, duration of hospitalization, and outcome. Short-term outcome was defined as outcome at the time of discharge from the hospital. The minimum long-term outcome was defined as outcome at 6 months post-discharge. This information was obtained by the surgeon or one of the authors (SAW) by using a telephone questionnaire.

## Results

### Horses

Eleven horses met the inclusion criteria. Their median age was 6 years (range 3 months to 21 years). The breeds included the American Quarter Horse (*n* = 8), Arabian (1), Thoroughbred (1), and Argentine Criollo (1). Four were mares, four were geldings, and three were stallions. The left TMJ of seven horses was affected, and the right TMJ of four horses was affected.

### History and Findings of Clinical Examination

Three horses had suffered trauma to the head (i.e., entrapment of the head in a fence, or hay feeder), but wounds had not been observed or had been deemed minor by the owner. The duration of signs of TMD prior to presentation ranged from 2 days to 270 days (median, 21 days; Table 1). Swelling in the region of the affected joint was the most common presenting complaint, but other complaints included difficulty eating, weight loss, hyporexia or anorexia, an acute or chronic wound in the region of the TMJ, and reluctance to accept the bit while being ridden. Eight horses displayed signs of pain when the region of the affected TMJ was palpated (Table 2), and only one horse was noted to have masseter muscle atrophy on the affected side.

**Table 1:** Signalment, duration of clinical signs, diagnosis, and prior therapy.

**Horse**	**Age** **(Years)**	**Breed**	**Sex**	**Side affected**	**Duration of clinical signs (Days)**	**Diagnosis**	**Therapy prior to UMC**
1	6	QH	MC	Right	270	Septic arthritis	IA steroid, Systemic ABs
2	3	QH	F	Left	6	Wound–Open Joint	Wound management, IA AB, systemic ABs
3	21	QH	M	Left	74	Osteoarthritis	Systemic ABs
4	11	QH	MC	Left	180	Osteoarthritis	None
5	2	QH	M	Right	1	Wound–Open Joint	Systemic ABs, joint lavage, IA ABs, sequestrum removal
6	0.25	QH	F	Left	14	Septic arthritis	Systemic ABs, arthrotomy, IA ABs
7	3	Arabian	F	Left	21	Septic arthritis	Mass debridement, systemic ABs
8	0.6	TB	M	Left	16	Septic arthritis	None
9	17	QH	MC	Right	21	Septic arthritis	Systemic ABs
10	6	AC	F	Right	1	Wound–Open Joint	Arthroscopic debridement, IA AB, systemic ABs
11	10	QH	MC	Left	90	Osteoarthritis	None

**Table 2:** Clinical signs on presentation.

	**Swelling^a^**	**Pain^b^**	**Difficulty eating**	**Bit issues**	**Weight loss**	**Wound**	**Facial n. paralysis**	**Masseter m. atrophy**	**Nasal discharge**
Horse 1	X	X	X		X				
Horse 2	X	X				X	X		
Horse 3	X		X						X^c^
Horse 4		X	X	X					
Horse 5		X	X			X			
Horse 6	X	X							
Horse 7	X	X						X	
Horse 8	X								
Horse 9	X	X	X						
Horse 10	X		X			X			
Horse 11	X								

a *Swelling over the TMJ*.

b *Pain on palpation of joint*.

c *Horse presented with concurrent guttural pouch empyema*.

### Diagnostic Imaging Results

**Figure 1:**
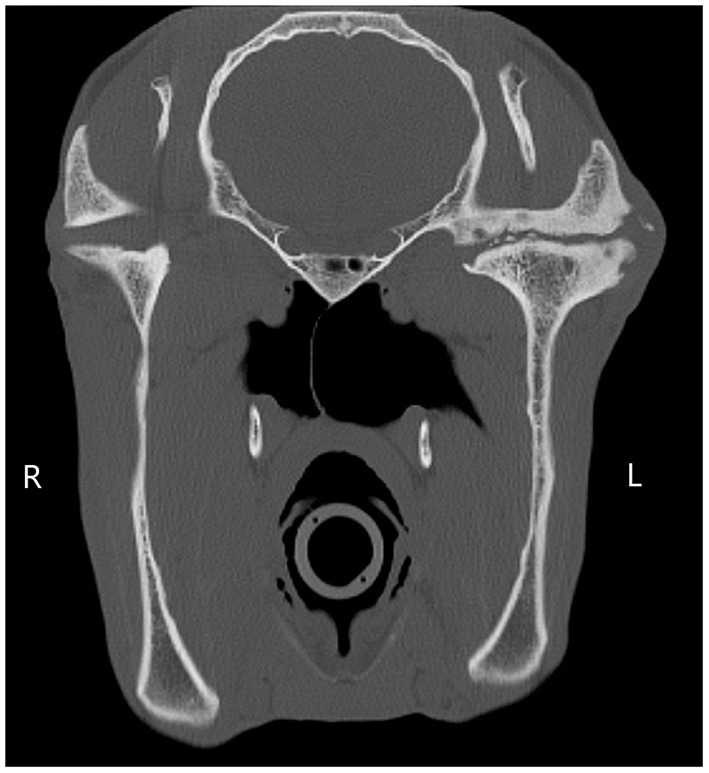
A transverse CT image showing soft tissue swelling, an irregular narrowed joint margin of the left mandibular condyle, multiple intra-articular osseous fragments, sclerosis and subchondral lucencies of the mandibular condyle and temporal bone. There is evidence of unilateral joint collapse, characterized by the reduced height of the vertical ramus of the left mandible compared to the right.

**Figure 2:**
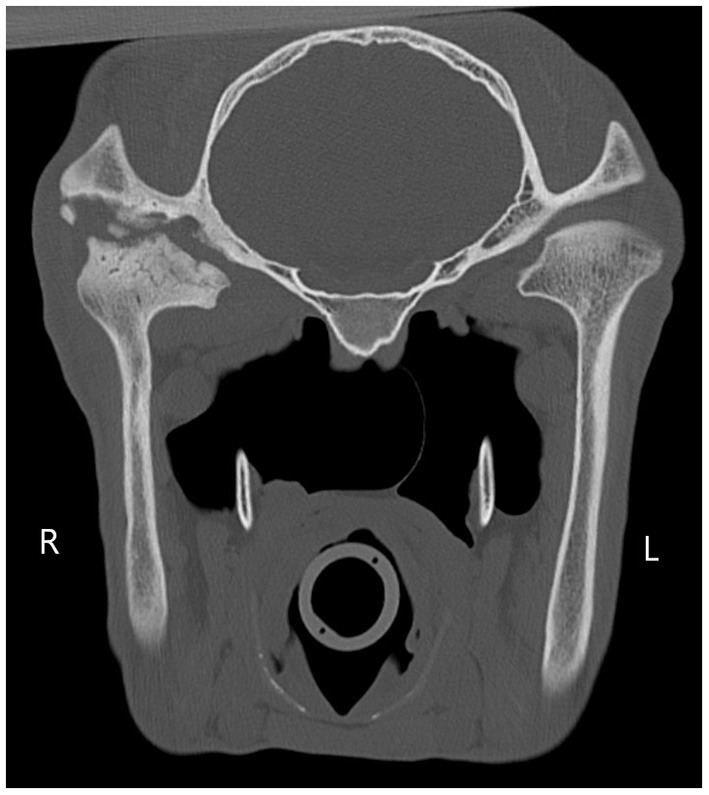
The right temporomandibular joint is widened. There is a large amount of multifocal lysis of the articular margins of the condylar process of the right mandible and right temporal bone, and to a lesser extent of the ventral aspect of the right zygomatic arch. There are multiple round osseous fragments surrounded by regions of hypoattenuation of the right temporal bone.

Not all horses received radiographic imaging of the skull, with or without tangential radiographic projections of the TMJ, however CT imaging was performed in all cases. Abnormal radiographic findings included an irregular lateral articular margin of the mandibular condyle, intra-articular osseous fragments, severe extra-capsular mineralization, and radiolucent areas within the mandibular condyle. One horse, for which no radiographic abnormalities of the TMJ were detected, was found, during CT imaging, to have lysis of the mandibular condyle, and sequestra within the joint. Other abnormal findings on CT examinations included articular fracture fragments (Figure 1), gas within the discotemporal compartment, despite no history of arthrocentesis, widening of the joint margin (Figure 2), subchondral erosions and sclerosis, fracture of the coronoid process, fracture of the zygomatic process of the temporal bone, and mild masseter and temporalis muscle atrophy. Four horses with TMD had CT evidence of concurrent disease of the ipsilateral temporohyoid articulation, characterized by thickening of the stylohyoid bone at the articulation. One of these four horses also had bilateral otitis media.

### Bacterial Culture and Sensitivity

Of the eight horses diagnosed with septic arthritis of the TMJ, six were confirmed to have sepsis of the joint based on bacterial culture of joint fluid obtained pre-operatively or from samples of bone removed at the time of surgery. One of the six had a polymicrobial bacterial infection, whereas a single bacterial isolate, including *Streptococcus equi ssp zooepidemicus*, methicillin-resistant or non-methicillin-resistant *Staphylococcus aureus, Rhodococcus equi, Actinobacillus sp*., or *Escherichia coli*, was cultured from the joint fluid or bone of the others. Two horses were presumed to suffer from sepsis of the joint due to the presence of a wound or draining tract that communicated with the joint.

### Surgical Technique

The surgical technique of condylectomy was consistent between surgeons and was similar to that described by others (13, 20, 23). The meniscus was removed from its capsular attachment using rongeurs or Mayo scissors. The cutaneous incision of eight horses was closed primarily, whereas the cutaneous incision of the remaining three was only partially sutured to allow for drainage of exudate or because of excessive tension on the incision. An active suction drain was placed into the cavity created by the condylectomy of three horses and removed 24–72 h post-operatively. All horses received appropriate pre- and post-operative antimicrobial and anti-inflammatory therapy.

### Complications

Only one horse had an intra-operative complication, which was the inadvertent transection of the transverse facial artery. The surgery was aborted and a pressure bandage was applied to the surgical site to stop the hemorrhage. A UMC was subsequently performed 2 months later.

Post-operative complications included serous or serosanguinous drainage from the incision (5 horses), swelling at the incision (5), formation of a sequestrum and draining tract at the incision (2), formation of an abscess ventral to the incision (1), signs of colic (2), and hyporexia (2) (Table 3).

**Table 3:** Post-operative complications.

**Post-operative complication**	**# Horses affected**
Incisional swelling/edema	5
Serous/serosanguinous discharge from incision	5
Abscess/fistula formation	3
Post-operative colic	2

Two horses experienced mild signs of post-operative colic (e.g., pawing, hyporexia, lying down). These signs of colic resolved within 24 h after periodic administration of a non-steroidal anti-inflammatory drug.

### Outcome

Horses remained hospitalized for a median of 7 days (range, 3 to 77 days) after surgery. One of the horses, a foal, had been hospitalized for 14 days for treatment for pneumonia caused by *Rhodococcus equi*, when it developed signs of sepsis of a TMJ, presumably caused by hematogenous spread of infection to the TMJ. This foal was hospitalized for 77 days for treatment for pneumonia and sepsis of the left femorotibial and femoropatellar joints and left TMJ. When this foal was excluded from analysis, the range of times horses were hospitalized was 3–30 days.

All horses were discharged alive from the hospital, and long-term follow-up ranged from 12 months to 8 years (Table 4). All 11 horses were able to consume a normal diet of hay and grain, and all returned to a normal grazing routine. Nine horses returned to athletic performance, one became a broodmare, and one continued to be used as a breeding stallion. Of the nine horses used for performance after surgery, one entered race training and had 22 starts over its 3-year racing career. One American Quarter Horse was in race training but had not yet raced at the time of publication. One horse won multiple World Championships in Western Pleasure or Working Ranch Horse shows. Six horses were used successfully for their intended function in Western Performance (e.g., barrel racing, team roping, working cow horse, western pleasure, and trail riding).

**Table 4:** Follow-up and intended use.

**Horse**	**Diagnosis**	**Duration of follow-up**	**Intended use**	**Return to work?**
1	Septic arthritis	39 months	Cutting/team penning/working ranch	Yes
2	Wound–Open Joint	42 months	Working cow horse	Yes
3	Osteoarthritis	47 months	Breeding stallion	Yes
4	Osteoarthritis	58 months	Barrel racing	Yes
5	Wound–Open Joint	26 months	Team Roping	Yes
6	Septic arthritis	29 months	QH Racehorse	Yes-In Training
7	Septic arthritis	108 months	Pleasure/Trail riding	Yes
8	Septic arthritis	42 months	TB Racehorse	Yes
9	Septic arthritis	95 months	Western Pleasure	Yes
10	Wound–Open Joint	12 months	Polo	No-Broodmare
11	Osteoarthritis	23 months	Pleasure	Yes^*^

* *Horse successfully returned to work but was later euthanized*.

In the two horses with mandibular displacement noted prior to surgery, post-operative oral examination revealed complete resolution in the lesser affected horse. The remaining horse had significant improvement but retained a permanent displacement of 1 cm and required biannual equilibration of the incisors and cheek teeth. The horse with the shear mouth had multiple follow-up oral examinations by a board-certified veterinary dentist, had normal excursion of the mandible to each side, but a mildly steeper occlusal angle to the cheek teeth on the side of the mandible that had undergone condylectomy.

A horse that was diagnosed with non-septic, degenerative osteoarthritis of the TMJ returned to pleasure riding after condylectomy, but was re-examined 2 years later due to acute, left-sided facial nerve paresis and head-shaking. Neurologic examination revealed the horse to be ataxic on all limbs and to have minor proprioceptive deficits characteristic of peripheral vestibular neuropathy. A marked enlargement and osteolysis of the stylohyoid bone at the temporohyoid articulation, characteristic of temporohyoid osteoarthropathy (THO), was identified during CT examination of the horse's head. The treatment recommended was a ceratohyoidectomy, but due to the severity of clinical signs and a guarded prognosis for complete resolution of clinical signs, the owner elected to have the horse euthanized. A necropsy confirmed the diagnosis of THO. A combination of woven bone and mature granulation tissue was found in the space of the previously excised condyle. The three other horses that had enlargement of the stylohyoid bone on CT did not display clinical signs consistent with THO in the follow-up period.

## Discussion

The findings of this retrospective study confirm the hypothesis that horses undergoing UMC, in combination with meniscectomy, as a treatment for severe disease of the TMJ can return to their previous or intended level of athletic function without the need for a specialized diet. All horses in this study, except for one mare, returned to their previous or intended use. This mare was considered capable of returning to its athletic use (polo), but was used instead as a broodmare due to her genetic value. No horse in this study developed mandibular drift, difficulty masticating, or ankylosis of the TMJ after UMC, all of which have been described in other species (25). These findings confirm that UMC should be considered a method of establishing good quality of life for horses with severe TMD and that UMC should not be considered solely a salvage procedure.

Whereas, fracture or subluxation of the mandibular condyle resulting from trauma has been reported to be the most common cause of disease of the TMJ (7), the majority of horses in this case series incurred damage to the TMJ from infection. Hematogenous spread of infection occurred in one foal, confirmed with bacterial culture from multiple joints, and idiopathic septic osteoarthritis was diagnosed in other horses.

Evidence of trauma to the head, combined with clinical signs of disease of the TMJ, such as difficult mastication, swelling or a wound in the region of the TMJ, and displacement of the mandibular incisors should prompt an examination of the TMJ. Early diagnosis and appropriate management of horses for arthritis of the TMJ, regardless of cause, may delay or eliminate the need for a UMC. All horses in this study had CT evidence of advanced degeneration of the TMJ, and some had been displaying signs of TMD for a substantial time (up to 270 days) prior to presentation at a referral hospital. Had subtle signs of infection of the TMJ been noted early in the course of disease, treatment by UMC may have been avoided (9). Horses with chronic osteoarthritis of the TMJ may have recognizable atrophy of the masseter muscles, osseous enlargement of the affected TMJ, weight-loss, malocclusion of the incisors and cheek teeth (13, 17, 18), which may prompt the clinician to consider the TMJ as the site of discomfort.

Difficulty in imaging the TMJ radiographically may also have contributed to delayed recognition of osteoarthritis. The ability to fully evaluate the nature and extent of TMD radiographically is limited, even with tangential projections, to the lateral third of the joint (26). The discotemporal and discomandibular joint compartments of the TMJ can be examined arthroscopically, and arthroscopic evaluation performed with the horse standing has been reported (14). Evaluation of the medial aspect of each compartment of the joint, however, can be technically challenging and requires mobility of the mandible (10, 27). Computed tomography, MRI, or nuclear scintigraphy may be more effective at recognizing disease of the TMJ (8, 17, 28, 29). Computed tomography of the equine head, in particular, has become more widely available and cost-effective. All 11 horses in this study received an examination using CT prior to surgery, and in each case, this examination accurately revealed the extent of disease of the TMJ and confirmed the necessity for treating the horse by UMC.

This retrospective study found that UMC was associated with few intra-operative and short-term post-operative complications. The only intra-operative complication was inadvertent transection of the transverse facial artery, illustrating that surgeons performing UMC must be aware of structures located in close proximity to the TMJ (1).

Elzer et al. (14) reported a horse that developed concomitant infection of a TMJ, otitis media, and THO (14). One horse in the current study, while being treated for empyema of both guttural pouches with orally administered antimicrobial drugs and daily lavage of the pouches, developed difficulty eating. This prompted CT examination of the head, which revealed that the horse had osteolysis of the mandibular condyle; bilateral THO, based on thickening of both stylohyoid bones at the temporohyoid articulation; and bilateral otitis media. Despite receiving orally administered antimicrobial drugs, and having resolved the guttural pouch empyema, osteolysis of the mandibular condyle progressed as evidenced by repeat CT examination of the head 35 days later. After UMC, the horse was able to return to its use as a breeding stallion. Follow-up conversations with the owner 4 years after UMC revealed that the horse displayed no signs of THO.

The left stylohyoid bone of another horse was observed during CT examination to be thickened at its temporohyoid articulation. The horse was re-examined 2 years later because it displayed progression of signs of THO. There is a close spatial relationship between the TMJ, the inner ear, the temporal bone, and the guttural pouches. Despite the proximity of these structures to each other, concomitant disease of these structures has rarely been reported. Our findings and those of others (14) suggest, however, that a horse suffering from disease of one of these structures may suffer from concomitant disease of one or both of the other structures.

The horse euthanized because it had severe signs of THO was found to have developed a pseudo-condyle composed of woven bone and mature granulation tissue. Horses and sheep have been reported to experience partial regeneration of the mandibular condyle after condylectomy (13, 16, 30, 31), and children < 12 years old are reported to have a great capacity to regenerate a mandibular condyle after condylectomy (32).

Long-term follow-up with owners indicated that horses undergoing UMC had a good prognosis for performing at their previous or intended level of performance. Horses were able to masticate normally, required no dietary changes to maintain weight, and those that returned to riding did not have any difficulty accepting the bit. Owners found the cosmetic appearance of horses with UMC acceptable.

## Conclusion

The results of this study show that horses that have undergone UMC are able to maintain a normal diet and return to their previous or intended level of athletic function. Limitations of this study include its retrospective nature and collection of data from multiple referral centers. Reliance on medical records and evaluation of horses by owners may have introduced error in reported data.

## Data Availability Statement

The original contributions presented in the study are included in the article/Supplementary Material, further inquiries can be directed to the corresponding author.

## Ethics Statement

Ethical review and approval was not required for the animal study because medical records of client-owned animals were used in this study. Written informed consent for participation was not obtained from the owners because consent for retrospective review of medical records was not required, consent for owner questionnaire was obtained when available.

## Author Contributions

SW contributed to study design, data collection, interpretation, and analysis, and manuscript preparation. FA, TH, NC, JS, and KO contributed to data collection, interpretation, and manuscript preparation. SB contributed to study concept and design, data collection, and analysis. CA contributed to study concept and design, data collection, and manuscript preparation. JC contributed to study concept and design, data interpretation, and manuscript preparation. All the authors contributed to manuscript revision, read, and approved the submitted version.

## Conflict of Interest

The authors declare that the research was conducted in the absence of any commercial or financial relationships that could be construed as a potential conflict of interest.

## Publisher's Note

All claims expressed in this article are solely those of the authors and do not necessarily represent those of their affiliated organizations, or those of the publisher, the editors and the reviewers. Any product that may be evaluated in this article, or claim that may be made by its manufacturer, is not guaranteed or endorsed by the publisher.

## Abbreviations

Abs: Antibiotics
AC: Argentinian Criollo
CT: Computed tomography
F: Female
IA: Intra-articular
M: Male
MC: Male castrated
MRI: Magnetic resonance imaging
OA: Osteoarthritis
QH: Quarter Horse
RBC: Red blood cell
TB: Thoroughbred
TMD: Temporomandibular joint disease
TMJ: Temporomandibular joint
WBC: White blood cell.
