# Supplementary material for: Long-Term Outcome of Horses Undergoing Unilateral Mandibular Condylectomy and Meniscectomy for Temporomandibular Joint Disease
Source: Front Vet Sci. 2022 May 2;9:898096. doi: 10.3389/fvets.2022.898096 (PMC9108769; doi:10.3389/fvets.2022.898096)
Supplement: Supplementary file 1 [file Table_1.docx]

**Follow- up Questionnaire for Owners**

**Date of response:___ _**

PATIENT NAME/Record information

How is your horse doing today (alive/dead/sold)? If they died how old was the horse and what was the date of passing? If sold, at what age?

Any incisional complications at home (infection) after surgery? If yes, what kind of complication? Did it need surgical correction? Did the horse need any medication and if so what kind?

Has the horse had any signs of difficulty eating/TMJ clicking or weight loss? What was the cause? What is the current diet?

Has the horse had a 2^nd^ surgery? If yes, when (date), what was found at surgery and where was the surgery?

What is/was the function of the horse and has the horse returned to its function? If the horse was in training or had never performed before surgery, how is the horse performing today?

Please grade your satisfaction with the outcome and treatment of the horse on a scale of 1 -10 (very poor to very satisfied)
